# RETRACTION: Amputation and Limb Salvage Following Endovascular and Open Surgery for the Treatment of Peripheral Artery Illnesses: A Meta‐Analysis

**DOI:** 10.1111/iwj.70207

**Published:** 2025-02-02

## Abstract

**RETRACTION:** WangG. , LiH. , ChenB. , GuoP. , and ZhangH. , “Amputation and Limb Salvage Following Endovascular and Open Surgery for the Treatment of Peripheral Artery Illnesses: A Meta‐Analysis,” International Wound Journal 20, no. 9 (2023): 3558–3566, 10.1111/iwj.14229.37328950 PMC10588360

The above article, published online on 16 June 2023, in Wiley Online Library (http://onlinelibrary.wiley.com/), has been retracted by agreement between the journal Editor in Chief, Professor Keith Harding; and John Wiley & Sons Ltd. Following an investigation by the publisher, all parties have concluded that this article was accepted solely on the basis of a compromised peer review process. The editors have therefore decided to retract the article. The authors did not respond to our notice regarding the retraction.

**RETRACTION:** G. Wang , H. Li , B. Chen , P. Guo , and H. Zhang , “Amputation and Limb Salvage Following Endovascular and Open Surgery for the Treatment of Peripheral Artery Illnesses: A Meta‐Analysis,” International Wound Journal 20, no. 9 (2023): 3558–3566, 10.1111/iwj.14229.37328950 PMC10588360

The above article, published online on 16 June 2023, in Wiley Online Library (http://onlinelibrary.wiley.com/), has been retracted by agreement between the journal Editor in Chief, Professor Keith Harding; and John Wiley & Sons Ltd. Following an investigation by the publisher, all parties have concluded that this article was accepted solely on the basis of a compromised peer review process. The editors have therefore decided to retract the article. The authors did not respond to our notice regarding the retraction.

